# Interference with work in fibromyalgia - effect of treatment with pregabalin and relation to pain response

**DOI:** 10.1186/1471-2474-12-125

**Published:** 2011-06-03

**Authors:** Sebastian Straube, R Andrew Moore, Jocelyn Paine, Sheena Derry, Ceri J Phillips, Ernst Hallier, Henry J McQuay

**Affiliations:** 1 Department of Occupational, Social and Environmental Medicine, University Medical Center Göttingen, Göttingen, Germany; 2 Pain Research, Nuffield Division of Anaesthetics, Nuffield Department of Clinical Neurosciences, University of Oxford, Churchill Hospital, Oxford, UK; 3 Spreadsheet Factory, Stratfield Road, Oxford, UK; 4 School of Human and Health Sciences, Swansea University, Swansea, UK

## Abstract

**Background:**

Clinical trials in chronic pain often collect information about interference with work as answers to component questions of commonly used questionnaires but these data are not normally analysed separately.

**Methods:**

We performed a meta-analysis of individual patient data from four large trials of pregabalin for fibromyalgia lasting 8-14 weeks. We analysed data on interference with work, inferred from answers to component questions of Fibromyalgia Impact Questionnaire (FIQ), Short Form 36 Health Survey, Sheehan Disability Scale, and Multidimensional Assessment of Fatigue, including "How many days in the past week did you miss work, including housework, because of fibromyalgia?" from FIQ. Analyses were performed according to randomised treatment group (pregabalin 150-600 mg daily or placebo), pain improvement (0-10 numerical pain rating scale scores at trial beginning vs. end), and end of trial pain state (100 mm visual analogue pain scale [VAS]).

**Results:**

Comparing treatment group average outcomes revealed modest improvement over the duration of the trials, more so with active treatment than with placebo. For the 'work missed' question from FIQ the change for patients on placebo was from 2.2 (standard deviation [SD] 2.3) days of work lost per week at trial beginning to 1.9 (SD 2.1) days lost at trial end (p < 0.01). For patients on 600 mg pregabalin the change was from 2.1 (SD 2.2) days to 1.6 (SD 2.0) days (p < 0.001). However, the change in days of work lost was substantial in patients with a good pain response: from 2.0 (SD 2.2) days to 0.97 (SD 1.6) days (p < 0.0001) for those experiencing >/= 50% pain improvement and from 1.9 (SD 2.2) days to 0.73 (SD 1.4) days (p < 0.0001) for those achieving a low level of pain at trial end (<30 mm on the VAS). Patients achieving both >/= 50% pain improvement and a pain score <30 mm on the VAS had the largest improvement, from 2.0 (SD 2.2) days to 0.60 (SD 1.3) days (p < 0.0001). Analysing answers to the other questions yielded qualitatively similar results.

**Conclusions:**

Effective pain treatment goes along with benefit regarding work. A reduction in time off work >1 day per week can be achieved in patients with good pain responses.

## Background

Fibromyalgia is a common chronic pain condition. It occurs in 1-2% of the population, more often in women than in men [1-3]. Chronic pain conditions such as fibromyalgia have profound effects on quality of life [4-6] and lead to interference with work and loss of productivity [7-13].

In clinical trials of treatments for chronic painful conditions - including trials in fibromyalgia - data on time off work and interference with work are typically collected as answers to component questions of commonly used questionnaires. Answers from these component questions are used to calculate overall scores of the questionnaires, but are not usually analysed separately and specifically with regard to work-related outcomes. Such an analysis - though not originally intended by the inventors of the questionnaires or the triallists who used them - nonetheless has the potential to provide useful insights into the outcomes associated with pain treatment: Do the patients with the greatest improvement in pain scores over the duration of the trial and those who have the lowest levels of pain at trial end also have the largest benefit with regard to work? How large is the benefit? Do patients with no or little pain improvement and high end of trial pain scores achieve any meaningful benefit regarding work?

We have recently addressed the question whether benefit in chronic pain trials across different domains of life occurs in the same patients, using a large set of individual patient data from randomised controlled trials (RCTs) of pregabalin in fibromyalgia [14]. We found that those patients with the largest pain improvement (difference in pain intensity at trial beginning vs. end) also had the largest benefit with regard to quality of life and time off work. The study presented here builds on our previous results. Using the same dataset we present new analyses specifically relating to work. We investigated time off work and interference with work in patients with fibromyalgia based on data from work-related component questions from commonly used questionnaires. Analyses were conducted by randomised treatment group (pregabalin at doses of 150-600 mg per day or placebo), pain improvement (comparing pain intensity at the beginning and the end of trials), and final (end of trial) pain state.

We analysed answers to work-related component questions from different questionnaires commonly used in chronic pain trials all addressing work but using different wording and assessment scales. Our aims were threefold: first, to illuminate the relationship between pain treatment, pain response, and work; second, to investigate the comparability of answers to work-related questions from different questionnaires; and, third, to provide a framework for future analyses of data on work-related outcomes collected in clinical trials in chronic painful conditions.

## Methods

We performed a meta-analysis of individual patient data from four large trials of pregabalin for fibromyalgia lasting 8-14 weeks, analysing data on interference with work, inferred from answers to component questions of Fibromyalgia Impact Questionnaire (FIQ), Short Form 36 Health Survey (SF-36), Sheehan Disability Scale (SDS), and Multidimensional Assessment of Fatigue (MAF), including "How many days in the past week did you miss work, including housework, because of fibromyalgia?" from FIQ. Analyses were performed according to randomised treatment group (pregabalin at 150-600 mg daily or placebo), pain improvement (comparing 0-10 numerical pain rating scale scores at trial beginning and end), and end of trial pain state (100 mm visual analogue pain scale [VAS]).

Pfizer Inc. provided Excel files containing individual patient data from four RCTs of pregabalin (Lyrica) in the treatment of fibromyalgia along with PDF files of the corresponding clinical trial reports. Pfizer study ID numbers of these trials and ClinicalTrials.gov identifier numbers (where available) were as follows:

• 1008-105 [15]

• A0081056, NCT00645398 [16]

• A0081077, NCT00230776 [17]

• A0081100, NCT00333866 [18]

An enriched enrolment randomised withdrawal trial, the "FREEDOM trial" [19], was not included in our analysis because it was of fundamentally different trial design [20].

Patients in the four included trials were at least 18 years old, fulfilling American College of Rheumatology classification criteria for fibromyalgia (1990 classification criteria) and with pain scores of ≥40 mm on the 100 mm VAS after stopping any relevant pain or sleep medication. Patients were randomised to receive pregabalin (150 mg, 300 mg, 450 mg, or 600 mg per day), or placebo, predominantly with a 2-week dose escalation phase followed by fixed dosing for up to 14 weeks of total trial duration. The minimum requirement for inclusion in this individual patient meta-analysis was that trials had to be both randomised and double blind.

Trial and patient characteristics are detailed in Additional file 1. In the four trials 2757 patients aged between 18 and 82 years were treated with pregabalin or placebo. More than 90% of trial participants were women. One trial lasted 8 weeks (trial 1008-105); the others lasted 13 or 14 weeks. Pregabalin doses of 300 mg (685 patients) and 450 mg (687 patients) were used in all four trials, 600 mg (564 patients) in three, and 150 mg (132 patients) in one trial; placebo was given to 689 patients. Trial quality was assessed using the Oxford Quality Scale [21]. Validity was scored using the Oxford Pain Validity Scale [22]. All trials obtained the maximum scores of 5/5 and 16/16 on these scales, indicating high trial quality and validity.

We analysed individual patient data on pain scores and time off work or interference with work obtained at the beginning of the trials (baseline measurements) and at the end of trials. Data on time off work or interference with work were compared

• according to randomised treatment group (placebo or pregabalin at doses of 150, 300, 450, or 600 mg per day),

• according to pain improvement, as described by the comparison of baseline numerical pain rating scores (scale 0-10) with end of trial numerical pain rating scores (assessed after 8 weeks of treatment in trial 1008-105 and after 12 weeks of treatment in the three other trials), and

• according to pain state at end of trial, as assessed by the VAS pain score on a 0-100 mm scale.

**Figure 1:**
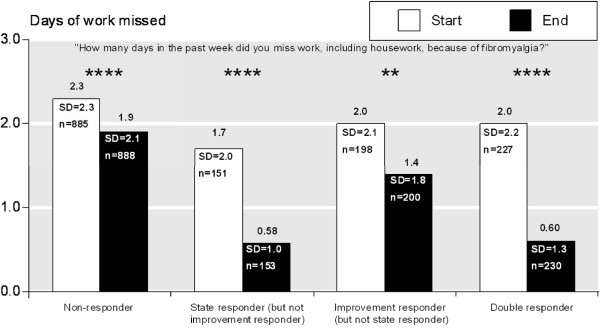
**Days of work missed**. Days of work missed per week at the beginning and end of trials as assessed by the question "How many days in the past week did you miss work, including housework, because of fibromyalgia?" from the Fibromyalgia Impact Questionnaire (FIQ). Data are presented as a comparison of 'state' and 'improvement' responders; 'non-responders' are neither state nor improvement responders; 'double responders' are both. ** p < 0.01, *** p < 0.001, **** p < 0.0001, NS - no significant difference, SD - standard deviation. FIQ data were not available for trial 105, the only trial with a 150 mg pregabalin group. Therefore no data for participants treated with 150 mg pregabalin are presented here.

**Figure 2:**
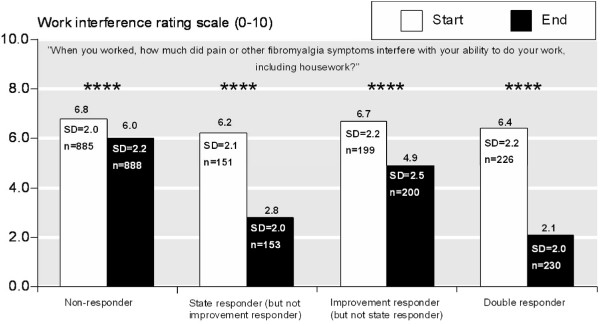
**Interference with work**. Interference with work as assessed by the question "When you worked, how much did pain or other fibromyalgia symptoms interfere with your ability to do your work, including housework?" from the Fibromyalgia Impact Questionnaire; responder categories and abbreviations as in Figure 1.

Not all questionnaires were used in all trials and not all patients had usable individual patient data available. Therefore the number of patients contributing to individual analyses differed. Numbers of patients contributing are given in Tables 1-2 and Figures 1-2.

**Table 1:** Days of work missed

	**Mean number (SD) of days of work missed per week at trial beginning**	**Mean number (SD) of days of work missed per week at trial end**	**Statistical significance**
**Pregabalin dose (n)**			
Placebo (557)	2.2 (2.3)	1.9 (2.1)	**
300 mg (547)	2.4 (2.3)	1.9 (2.1)	****
450 mg (553)	2.1 (2.2)	1.7 (2.0)	***
600 mg (561)	2.1 (2.2)	1.6 (2.0)	***
**Pain improvement (n)**			
Pain worse (221)	1.9 (2.1)	1.9 (2.1)	NS
0-15% (281)	2.5 (2.4)	2.2 (2.2)	NS
15-30% (233)	2.4 (2.2)	1.7 (2.0)	***
30-50% (319)	2.1 (2.2)	1.1 (1.7)	****
≥50% (435)	2.0 (2.2)	0.97 (1.6)	****
**VAS pain score at trial end (n)**			
>50 mm (1042)	2.4 (2.3)	2.1 (2.1)	*
>30-50 mm (340)	1.9 (2.0)	1.1 (1.7)	****
0-30 mm (436)	1.9 (2.2)	0.73 (1.4)	****

Days of work missed per week at the beginning and end of trials as assessed by the question "How many days in the past week did you miss work, including housework, because of fibromyalgia?" from the Fibromyalgia Impact Questionnaire (FIQ). Data are presented according to daily pregabalin dose, according to improvement in pain intensity scores over the course of the trials, and according to pain state at end of the trials; statistical significance for the comparison of trial end vs. beginning: * p < 0.05, ** p < 0.01, *** p < 0.001, **** p < 0.0001, NS - no significant difference. FIQ data were not available for trial 105, the only trial with a 150 mg pregabalin group. Therefore no data for participants treated with 150 mg pregabalin are presented here.

**Table 2:** Interference with work

	**Mean score (SD) at trial beginning**	**Mean score (SD) at trial end**	**Statistical significance**
**Pregabalin dose (n)**			
Placebo (556)	6.8 (2.1)	5.6 (2.6)	****
300 mg (551)	6.7 (2.1)	5.5 (2.6)	****
450 mg (553)	6.7 (2.1)	5.3 (2.6)	****
600 mg (562)	6.7 (2.1)	5.5 (2.7)	****
**Pain improvement (n)**			
Pain worse (221)	6.3 (2.2)	6.1 (2.2)	NS
0-15% (282)	7.2 (1.9)	6.5 (2.2)	****
15-30% (233)	6.8 (2.1)	5.7 (2.3)	****
30-50% (319)	6.6 (1.9)	4.2 (2.3)	****
≥50% (435)	6.6 (2.2)	3.5 (2.6)	****
**VAS pain score at trial end (n)**			
>50 mm (1046)	7.0 (2.0)	6.5 (2.1)	****
>30-50 mm (326)	6.3 (2.1)	4.5 (2.0)	****
0-30 mm (387)	6.3 (2.2)	2.8 (2.3)	****

Interference with work as assessed by the question "When you worked, how much did pain or other fibromyalgia symptoms interfere with your ability to do your work, including housework?" (scale 0-10) from the Fibromyalgia Impact Questionnaire. Data are presented according to daily pregabalin dose, according to improvement in pain intensity scores over the course of the trials, and according to pain state at end of the trials; abbreviations as in Table 1.

Cut-off levels for pain improvement and end of trial pain state were chosen with clinical relevance in mind and were based on published evidence. The Initiative on Methods, Measurement, and Pain Assessment in Clinical Trials (IMMPACT) defined benchmarks for interpreting change in chronic pain clinical trial outcome measures [23]: a 'minimally important' improvement was defined as a 10-20% decrease in pain intensity, a 'moderately important' improvement was defined as at least 30% decrease, and a 'substantial' improvement equated to at least 50% decrease in pain intensity. This formed the basis for our approach to classify improvement in pain intensity as 0-<15%, 15-<30%, 30-<50%, ≥50%, or worsening. Furthermore, pain greater than about 30 mm on a 100 mm VAS is equivalent to 'moderate' or greater pain [24,25] and therefore a pain level below 30 mm appears to be a suitable clinical target. Hence 0-30 mm was chosen as the strictest category of end of trial pain state and was compared with the pain states >30-50 mm and >50 mm.

We also analysed time off work and interference with work according to whether patients achieved the status of a 'state responder' (end of trial VAS pain score of 30 mm or less), 'improvement responder' (at least 50% improvement in numerical pain rating scale score over the duration of the trials), 'double responder' (state and improvement responder), or 'non-responder' (neither state nor improvement responder).

This approach was applied to the following five component questions from four commonly used questionnaires:

• "How many days in the past week did you miss work, including housework, because of fibromyalgia?" (scale 0-7) from the FIQ;

• "When you worked, how much did pain or other fibromyalgia symptoms interfere with your ability to do your work, including housework?" (scale 0-10) from the FIQ;

• "During the past 4 weeks, how much did pain interfere with your normal work (including both work outside the home and housework)?" (scale 1-5) from the SF-36;

• "The symptoms have disrupted your work/school work" (scale 0-10) from the SDS;

• "In the past week, to what degree has fatigue interfered with your ability to work?" (scale 1-10) from the MAF.

Spreadsheet calculations from the individual patient data were performed independently of Pfizer by one of the authors: Jocelyn Paine from the commercial spreadsheet calculating and programming service 'Spreadsheet Factory' [26]. The statistical significance of differences in mean values at the beginning and end of trials was established with the t test using 'QuickCalcs' [27].

## Results

### Days of work lost per week

Based on response data from the question "How many days in the past week did you miss work, including housework, because of fibromyalgia?" from the FIQ we compared placebo and pregabalin treatment arms of the trials (Table 1). There was statistically significant improvement at trial end (compared with the baseline values at trial beginning) in all treatment arms, including in the patients treated with placebo, though greater improvement occurred with pregabalin treatment.

While the analysis of patients according to randomised treatment group (and irrespective of pain response) reveal only modest average improvements when trial beginning and end were compared, analysing patients according to pain improvement revealed substantial differences. Those patients with the largest pain improvements (changes in 0-10 numerical pain rating scale score from trial baseline to trial end) also had the largest changes in days off work. Those with at least 50% pain improvement gained about one day of work per week (Table 1).

Analysing days off work by end of trial pain state (100 mm VAS score) likewise revealed a close relationship between pain experience and time off work. While there was little difference in time off work in those with a final VAS pain state >50 mm, those with a pain state >30-50 mm benefited substantially, and patients with a final pain state of 0-30 mm benefited extensively, gaining about 1.3 days of work per week (Table 1).

Next we analysed time off work according to status as a 'state responder', 'improvement responder', 'double responder' or 'non-responder' (Figure 1). Patients who were neither state nor improvement responders had the least benefit in term of gaining time at work. Patients who were improvement but not state responders benefited more and those who were state but not improvement responders had even more benefit. Double responders had the largest benefit, with an average of 1.4 more days of work per week

### Interference with work as assessed from the FIQ

We analysed answers to the question "When you worked, how much did pain or other fibromyalgia symptoms interfere with your ability to do your work, including housework?" from the FIQ in an analogous manner to answers to the question about days of work missed. Our findings were similar: Analysing all patients independently of pain outcomes there was improvement with regard to work over the duration of the trials in every treatment group, more so with pregabalin than with placebo, though the differences were not large. Those with the most pain improvement and the least end of trial pain scores had the best outcome in terms of experiencing less interference with work, while those with little pain improvement and high end of trial pain scores had little benefit (Table 2). Double-responders had the largest benefit with a 67% reduction in average work interference, followed by state only responders (55%), improvement only responders (27%), and non-responders (12%; Figure 2).

### Other questions about interference with work

The answers to the component questions about interference with work from the other questionnaires produced a similar picture. Categorising patients by randomised treatment group (pregabalin 150, 300, 450, or 600 mg or placebo) revealed modest improvement when trial beginning and end were compared, somewhat more so in the pregabalin groups than with placebo (data not shown).

Analyses of state and improvement responders likewise yielded qualitatively similar results. We investigated between-question similarity by calculating the percent improvement in answers to the different work-related questions, in each case comparing responses at trial beginning and end. We performed these analyses according to pain improvement over the duration of the trials (Table 3) and according to end of trial pain state (Table 4). For example, patients in the category of ≥50% improvement in pain score had a 52% improvement in the score for the FIQ question about days of work missed and 47% improvement in work interference as assessed from the FIQ question; those in the category of 30-50% pain improvement had an improvement in the answers to those questions of 47% and 36% respectively. Lower levels of pain benefit were associated with smaller reductions in work interference, and patients without pain improvement had no meaningful improvement either with regard to days of work missed or with regard to interference with work in any question (Table 3). The pattern of higher levels of pain intensity reduction being associated with greater reductions in work interference was found for questions from all four questionnaires (FIQ, SF-36, SDS, and MAF). The degree of benefit was similar in the three questionnaires using a 0-10 or 1-10 scale (FIQ, SDS, and MAF), with somewhat less benefit for SF-36, using a five-point scale.

**Table 3:** Improvement in answers to work-related questions according to pain improvement

**Pain improvement**	**FIQ (days of work missed)**	**FIQ (interference with work)**	**SF-36 (interference with work)**	**SDS (interference with work)**	**MAF (interference with work)**
≥ **50%**	52	47	33	62	57
**30 - <50%**	47	36	23	42	35
**15 - <30%**	28	16	16	28	28
**0 - <15%**	12	10	8.2	12	0.95
**<0%**	-1.0	2.8	1.8	-6.9	-10

Percent improvement in answers to work-related questions over the duration of the trials; patients were categorised by pain improvement (based on the 0-10 numerical pain rating scale comparing trial beginning and end); FIQ - Fibromyalgia Impact Questionnaire, SF-36 - Short Form 36 Health Survey, SDS - Sheehan Disability Scale, MAF - Multidimensional Assessment of Fatigue.

**Table 4:** Improvement in answers to work-related questions according to pain state

**Pain state (VAS score)**	**FIQ (days of work missed)**	**FIQ (interference with work)**	**SF-36 (interference with work)**	**SDS (interference with work)**	**MAF (interference with work)**
≤ **30 mm**	61	56	41	68	58
**30 - 50 mm**	41	28	22	46	37
**> 50 mm**	9.9	7.1	5.9	12	6.2

Percent improvement in answers to work-related questions over the duration of the trials; patients were categorised according to pain state (0-100 mm VAS score) at the end of trial; FIQ - Fibromyalgia Impact Questionnaire, SF-36 - Short Form 36 Health Survey, SDS - Sheehan Disability Scale, MAF - Multidimensional Assessment of Fatigue.

Patients with an end of trial pain state of 30 mm or less on the 0-100 mm VAS had an improvement in the score for the FIQ questions about days of work missed and work interference of 61% and 56%, respectively. Those with a final VAS score >50 mm only had minimal improvement in days of work lost and work interference (Table 4). Again, less pain was associated with greater improvement in work interference for all questions, and the degree of benefit was similar in the three questionnaires using a 0-10 or 1-10 scale (FIQ, SDS, and MAF), with somewhat less apparent benefit for SF-36 where a five-point scale was used.

## Discussion

Analysis of data at the level of the individual patient can reveal insights over and above those that can be obtained from the analysis of group mean values but it is only possible when trial data are made available at the level of the individual patient. We had such data for pregabalin trials in fibromyalgia, but not for pregabalin in other painful conditions, nor for other drugs in fibromyalgia or other painful conditions. The availability of clinical trial data at the level of the individual patient is uncommon, especially where trials have consistently used several different assessment scales permitting analysis of the relationship between treatment success as measured by pain relief and work interference.

This study demonstrated that most benefit regarding work in trials of pregabalin for fibromyalgia is obtained by patients who have large improvements in pain intensity, achieve a low level of pain after treatment, and especially if they achieve both of these outcomes. Comparing treatment groups irrespective of pain outcomes reveals a picture of only modest average benefit of active treatment when compared with placebo. However, substantial benefit in work-related outcomes can be demonstrated in those for whom treatment 'works' in the sense that it leads to a reduction in pain or a low end of trial pain state. Relationships between higher levels of pain and increased disability and interference with activities have been shown for a variety of conditions including cancer pain, phantom limb pain, back pain, and carpal tunnel syndrome [28-30], but improvements in work-related endpoints on return to a low pain state are less well described.

The point that fibromyalgia leads to substantial interference with work is well worth making. The status of fibromyalgia as a valid disease entity, let alone one with a substantial impact on a variety of domains of life, has long been in question because a clear physical correlate of the disease could not be demonstrated. Not surprisingly, in the face of such scepticism the diagnosis of fibromyalgia is often delayed [13]. Recently, however, evidence pointing to objective changes is accumulating [31]. A link between elevated intrinsic brain connectivity and spontaneous pain intensity in fibromyalgia has been shown [32], as has an association between cerebral blood flow and response to treatment with gabapentin [33]. Along with the emerging evidence of a physical correlate comes the insight that fibromyalgia has real and profound impact across different domains of life including work [8-10,12,13] and that the economic burden associated with fibromyalgia is substantial, also because of a high prevalence of co-morbidities [34]. Disease state and interference with work are linked in fibromyalgia, as the present and other studies demonstrate. Temporary work disability in fibromyalgia patients has been associated with a worse clinical situation and worse functional capacity [35]. A narrative interview study uncovered that severe pain and fatigue, together with a demanding life situation and ageing, seemed to cause substantial interference with work and functioning [36]. Remaining at work despite fibromyalgia requires 'a constant struggle' against the symptoms and consequences of the disease [37]. Increased awareness of fibromyalgia as a genuine condition along with practice-oriented diagnostic criteria and a scale for the assessments of symptom severity [38], will hopefully lead to more prompt diagnosis and adequate treatment.

The IMMPACT group suggested that 50% improvement in pain intensity is 'substantial improvement' [23]. Other evidence suggests that 30 mm is a suitable cut-off to determine a target pain state [24,25]. Our findings on time off work and interference with work underline the usefulness of these suggested cut-offs in clinical practice.

Our analysis provides further evidence that benefit due to treatment of fibromyalgia with pregabalin across different domains of life occurs largely in the same patients, as we previously suggested [14]. Those patients with the lowest end of trial pain levels and the most pain improvements had the largest benefits regarding work. This underlines the point that effective pain treatment should be sought, both from a patient perspective and also from an economic perspective [39], and that pain outcomes are linked with outcomes in other domains of life. Achieving the state of effective pain control will probably involve changing therapy in a substantial number of patients because no therapy works well in more than half the patients with fibromyalgia [40-42]. Therefore, always using just one drug for treating this complex and varied condition, even if it appears best on a population level, is unlikely to deliver the best outcomes for the most patients.

We found that data from several questions about time off work and interference with work with somewhat different wording and from different questionnaires yielded overall similar results, though when expressed as percent improvement in scores (comparing trial beginning and end, Tables 3 and 4) there were differences in the magnitude of the improvement. Caution is therefore needed when data from different work-related questions are compared. The preliminary findings here would indicate that questionnaires with 0-10 or 1-10 scales measuring work interference are associated with bigger improvements in work interference measures than a questionnaire with a 1-5 scale. This may be an effect of the scales, because the maximum possible reduction that can be attained on a 1-5 scale is 80%, while it is 90% on a 1-10 scale and 100% on a 0-10 scale.

Despite the large number of patients studied in four high quality randomised trials, the analysis presented here has important limitations, not least being a retrospective analysis that is only hypothesis-generating. As mentioned previously, using component questions of questionnaires in this way was not intended by the developers of the questionnaires, and the questionnaires have therefore not been validated for this use. Furthermore, the wording of the questions addressing 'interference with work' does not allow a differentiation into what kind of 'interference' (absenteeism, presenteeism) is present. We had no objective records of time at work or time off work on which to base our analysis; nor did we have a detailed description of the type of occupation. This would have been interesting because different kinds of occupation may be differently affected by pain and its treatment. Moreover, we only used data from four trials of pregabalin in fibromyalgia where we had individual data available; other chronic pain conditions and treatments might produce different results.

These limitations determine the agenda for future research: The first step would be a repetition of this type of analysis for different work-related questions, different treatments and different chronic pain conditions to determine whether or not our findings are general. Because data on time off work and interference with work are commonly collected in chronic pain trials, this is primarily a question of obtaining access to the data and performing the analyses. The second step will be more difficult: to collect more detailed information on occupation and objective data on time off work as part of clinical trials in chronic pain. We hope that raising the awareness of these issues will lead to such data being collected in chronic pain trials in the future.

## Conclusions

This meta-analysis of individual patient data from four trials of pregabalin for fibromyalgia demonstrates that effective pain treatment, described in terms of pain improvement over the course of the trials and end of trial pain state, is linked with benefit regarding work. A reduction in time off work of more than one day per week can be achieved in patients with good pain responses. Analysing answers to work-related component questions from different questionnaires yields qualitatively similar results.

## Abbreviations

FIQ: Fibromyalgia Impact Questionnaire; IMMPACT: Initiative on Methods, Measurement, and Pain Assessment in Clinical Trials; MAF: Multidimensional Assessment of Fatigue; NS: no significant difference; RCT: randomised controlled trial; SD: standard deviation; SDS: Sheehan Disability Scale; SF-36: Short Form 36 Health Survey; VAS: visual analogue scale

## Competing interests

SS received research support and grants from charities, Bandolier, Reckitt Benckiser, and Georg-August-University Göttingen and received lecture fees from University Children's Hospital Zurich and Oxford Medical Knowledge. RAM received lecture fees from Merck, Pfizer, Menarini, Lundbeck, AstraZeneca; served as a consultant for Merck, Pfizer, Eli Lilly, Flynn, Futura, Altana, Fujisawa, GSK, Shire, Sanofi-Aventis, Grünenthal, Organon, ZARS; received research grants from Merck, Pfizer, Abbott, AstraZeneca, Menarini, GSK, Aspreva, Janssen-Cilag. CJP received lecture fees from Merck, Gilead and Lundbeck in the past year, served as a consultant for Merck, Pfizer and Sanofi-Aventis in the past year and received a departmental grant from Napp in the past year. SD received research support from UK government and charities. HJM received lecture or consultancy fees in the past year from Menarini, Esteve, Grünenthal, Pfizer, Reckitt Benckiser, IMS and Archimedes.

## Authors' contributions

SS and RAM were involved with the original concept, planning the study, writing it, analysis, and preparing a manuscript; JP performed the calculation; SD, CJP, EH, and HJM. were involved with planning, and writing. All authors read and approved the final manuscript.

## Pre-publication history

The pre-publication history for this paper can be accessed here:

http://www.biomedcentral.com/1471-2474/12/125/prepub

## Supplementary Material

Additional file 1 **Study and patient characteristics**. The studies are identified by their Pfizer study ID numbers, the ClinicalTrials.gov identifier numbers (where available), and references to publications. Click here for file
