# Supplementary material for: Interference with work in fibromyalgia - effect of treatment with pregabalin and relation to pain response
Source: BMC Musculoskelet Disord. 2011 Jun 3;12:125. doi: 10.1186/1471-2474-12-125 (PMC3118156; doi:10.1186/1471-2474-12-125)
Supplement: Additional file 1 — Study and patient characteristics. The studies are identified by their Pfizer study ID numbers, the ClinicalTrials.gov identifier numbers (where available), and references to publications. [file 1471-2474-12-125-S1.PDF]

Additional file 1 - Study and patient characteristics

The studies are identified by their Pfizer study ID numbers, the ClinicalTrials.gov identifier numbers (where available), and references to publications.

Study and patient characteristics	Trial 1008-105 [15]	Trial A0081056, NCT00645398 [16]	Trial A0081077, NCT00230776 [17]	Trial A0081100, NCT00333866 [18]
**Total patients**	530 (randomised)<br>529 (received study medication)	751 (randomised)<br>748 (received study medication)	750 (randomised)<br>745 (received study medication)	747 (randomised)<br>735 (received study medication)
**Female sex**	485 (91.5%)	706 (94.4%)	704 (94.5%)	672 (91.4%)
**White ethnicity**	494 (93.2%)	675 (90.2%)	678 (91.0%)	558 (75.9%)
**Age: mean (SD), range**	48.6 (10.6)<br>20-78	48.8 (10.9)<br>18-82	50.1 (11.4)<br>18-81	48.5 (11.2)<br>20-81
**Duration of fibromyalgia in months: mean (SD), range**	107.7 (100.5)<br>0-654	111.7 (95.0)<br>3-656	120.2 (96.2)<br>1-614	98.8 (93.9)<br>3-554
**Study centres**	40 centres in the USA	79 centres in the USA	85 centres in the USA	72 centres in Canada, Mexico, Venezuela, Denmark, France, Germany, Italy, Netherlands, Portugal, Spain, Sweden, Switzerland, United Kingdom, India, Republic of Korea, and Australia
**Study duration (weeks)**	8	13	14	14
**Treatment groups: pregabalin doses per day (n)**	Placebo (n=131)<br>150 mg (n=132)<br>300 mg (n=134)<br>450 mg (n=132)	Placebo (n=190)<br>300 mg (n=185)<br>450 mg (n=183)<br>600 mg (n=190)	Placebo (n=184)<br>300 mg (n=183)<br>450 mg (n=190)<br>600 mg (n=188)	Placebo (n=184)<br>300 mg (n=183)<br>450 mg (n=182)<br>600 mg (n=186)
